# Thalamic Abscess in a Newly Diagnosed Diabetic Patient

**DOI:** 10.1155/2019/2719505

**Published:** 2019-11-07

**Authors:** Kenneth A. Swanson, Robert J. Citronberg

**Affiliations:** ^1^Advocate Lutheran General Hospital, Internal Medicine, 1775 Ballard Road, Park Ridge, Illinois 60068, USA; ^2^Infectious Diseases Department Chair, Advocate Lutheran General Hospital, Northwest Infectious Disease Consultants, 7900 North Milwaukee Avenue, Suite 231-A, Niles, Illinois 60714, USA

## Abstract

Brain abscesses are commonly located in the cerebral cortex and cerebellum; however, solitary thalamic lesions are rare. Recent cases report dental pathology as a common source, potentially compounded by immunocompromise. Here, we report a newly diagnosed diabetic male with poor dentition presenting with evidence of meningitis wherein thalamic abscess was discovered.

## 1. Case Report

**Figure 1:**
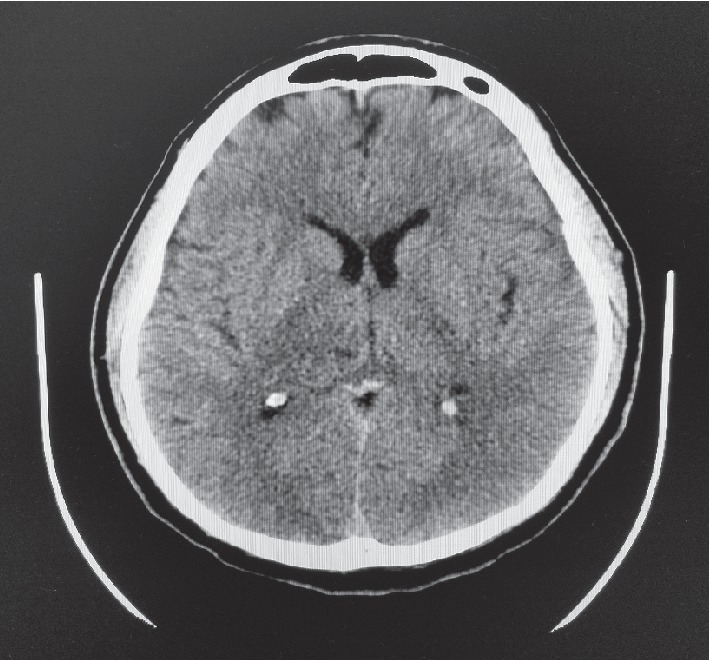
CT brain without contrast interpreted as normal.

**Figure 2:**
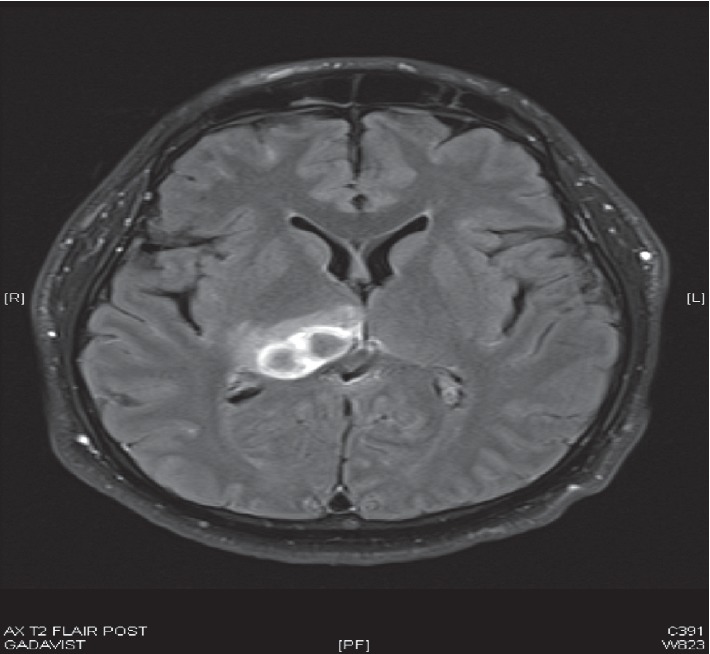
T2 flair MRI brain with contrast showing a 3 cm multiloculated cystic lesion (abscess) over the posterior thalamus with surrounding edema.

A 38-year-old man presented to a community hospital complaining of 5 days of gradually worsening headache and fatigue with associated vomiting, neck stiffness, photophobia, sweats, and chills. He had been seen at immediate care and was presumed to have influenza. The morning of presentation, the patient experienced altered mental status and was brought by his family to the emergency department. He was febrile at 39.4°C and tachycardic to 110 BPM with somnolence. Neurological examination was nonfocal, but positive nuchal rigidity and poor dentition were noted. Blood cultures x2 were drawn. Labs were remarkable for elevated fasting glucose of 318, procalcitonin-2.38, and WBC-19. Urinalysis was normal. CT head without contrast showed no abnormalities (Figure 1). Lumbar puncture CSF studies showed xanthochromia with glucose-133, protein-642, RBCs-295, and nucleated cell count-12,000 with differential: segs-72, monocytes-25, lymphocytes-2.0, basophils-1, and eosinophils-0. The infectious diseases service was consulted on suspicion of bacterial meningitis. CSF viral panel and culture were obtained, and then he was started on empiric vancomycin, ceftriaxone, and dexamethasone as well as one dose of acyclovir. In the subsequent 3 days, he was diagnosed with diabetes mellitus (DM) based on hemoglobin A1c of 10.9, mental status improved significantly, and sepsis subsided. Respiratory and CSF viral panels were negative. CSF culture showed no growth, and West Nile CSF IgM was negative. WBC count downtrended to a normal level of 11 by day 4 of admission, and vancomycin and dexamethasone were discontinued with plans to have PICC placed for 2 weeks of ceftriaxone alone. On day 5, he worsened with headaches, fever to 39.2°C, nausea/vomiting, and worsening leukocytosis with WBC count of 12.1. Vancomycin was restarted. White blood cell count rose to 13.2. Both transthoracic and transesophageal echocardiograms did not find vegetations. MRI brain (Figure 2) and C-spine were ordered to exclude parameningeal focus of infection. This revealed a multiloculated cystic lesion representing a 3 cm abscess centered over the posterior right thalamus with adjacent meningitis. No ventriculitis or ependymitis was appreciated. Chest X-ray was unremarkable. IV.

Metronidazole was added. Neurosurgery was consulted. Stereotactic aspiration procedure was not recommended due to high risk and relatively low chance of microbial diagnosis. Continuing antibiotic therapy was recommended with plans to repeat MRI brain in one week. Repeat CSF showed no growth. By day 9, WBCs normalized to 7.5, and symptoms improved but low-grade fevers around 38.2–38.4°C persisted. Repeat MRI brain one week later showed no improvement. Fevers again climbed as high as 39.6°C, and neurosurgery again determined drainage to be too risky. Ceftriaxone and metronidazole were discontinued. Meropenem was started and vancomycin was continued. His serum glucose became well-controlled with the use of basal bolus and prandial insulin. On day 9, he was transferred to a university hospital where MRI demonstrated increasing reach of the infectious material to the posterior third ventricle and into the contralateral thalamus, and again no ventriculitis or ependymitis was appreciated. The following day, the patient was taken to the operating room for neurosurgical intervention. An entry point just above the transverse sinus in the temporal occipital region was planned. A single bur hole was made, and a needle was inserted along the preplanned trajectory. Evacuation via biopsy syringe revealed frank pus. The procedure was tolerated without complication. No identifiable organism was isolated in the aspirate despite ribosomal sequencing. Dentistry was consulted and evaluated for dental infection and found no evidence of abscess or periodontitis. He was discharged on IV vancomycin and ampicillin-sulbactam for four additional weeks to complete a total antibiotic course of six weeks. Significant improvement was observed on repeat MRI four weeks and then twelve weeks after discharge. He was followed closely as an outpatient during this time and beyond.

## 2. Discussion

Thalamic brain abscesses are quite rare, making up 2.2–4.5% of reported brain abscesses [1, 2]. Most brain abscesses occur in the cerebral cortex, and in 20–40% of patients no etiology is discovered [3, 4, 5]. Thalamic abscess carries a mortality rate of 9–14% when rupture into the ventricles has not taken place [2, 6]. However, mortality approaches 80% if there is rupture [1]. The most common reported causative microbes are anaerobes and *Streptococci*, although 28% of thalamic abscess cases reported are culture-negative [7]. The causative pathogen was never identified in our patient.

Of note, the patient was diabetic with poor dentition. One 2014 case report describes a middle-aged gentleman with previously unknown DM with hemoglobin A1c of 10.7 and an asymptomatic periodontitis resulting in a thalamic abscess positive for *Streptococcus milleri*. He was treated with aspiration and six weeks of antibiotics with tooth extraction three months later [8]. A 2015 case report describes another middle-aged man with DM who was diagnosed with thalamic abscess due to periodontal disease which was discovered on CT imaging. In the latter case, the causative pathogen was *Streptococcus intermedius* identified via aspiration but unfortunately died [9]. A third case reported a significantly malnourished 25-year-old man with thalamic abscess and a piece of subcutaneous shrapnel on the ipsilateral side of the head, although causation could not be proven. *Streptococcus constellatus* was grown in aspirate culture and successfully treated with a twelve-week antibiotic course [10]. All three of these cases have immunocompromise in common with odontogenic bacteria grown in culture. Two of them specifically were diabetics with dental infections while one was immunocompromised due to malnourishment. Chronic periodontal disease and type 2 DM are proposed to be interrelated, but both conditions are common while thalamic abscess is uncommon [8]. Causation can be difficult to prove. A 2006 publication proposed that odontogenic brain abscess should meet the following 3 criteria: (1) alternative source of bacteremia is not apparent, (2) pathogens should be consistent with oral microflora, and (3) there should be dental pathology present on physical or radiographic exam [11]. In this case, these criteria were not met. While he eventually underwent specialist dental examination and dental X-ray at the university hospital which yielded no visible infectious process, he never had dedicated CT scans of the mandible and maxilla. Other common sources such as endocarditis, pulmonary, and sinusitis were excluded early on. Additionally, preantimicrobial blood cultures showed no growth. Therefore, it is not possible to definitively diagnose or exclude a dental or periodontal source of abscess in our patient retrospectively. In the absence of source identification, our patient was successfully treated with six weeks of antibiotics. Four to six weeks of antibiotic therapy has demonstrated efficacy in patients suffering from brain abscesses treated with drainage and six to eight weeks in those who did not undergo drainage [12]. However, recurrence of brain abscesses in patients receiving less than three weeks of antibiotics was described in a retrospective study [13].

In summary, this young patient had poor dentition and newly diagnosed diabetes mellitus with a single thalamic brain abscess of uncertain origin. Contrast-enhanced imaging is not routinely performed when patients present with meningitis, so it is unclear how often an abscess goes undetected, especially one in such an uncommon location easily missed by head CT without contrast. In our patient, no clear lesion was identified on noncontrast CT. Given that the patient presented with altered mental status manifesting as somnolence, MRI with contrast would likely have been beneficial earlier to rule out complications such as abscess, ventriculitis, cerebritis, or subdural empyema [14]. We propose that in immunocompromised and poorly controlled diabetic patients with suspected CNS infection, especially those with neurologic sequelae such as altered mental status, a low threshold should be kept for obtaining contrast-enhanced brain imaging early on to rule out abscess. Additionally, a high index of suspicion should be kept for odontogenic and/or periodontal sources of thalamic abscess if another source is not found.
